# From Pieces to Processes: Teaching Health Care Process Optimization and Quality Improvement Through a Building Brick Simulation

**DOI:** 10.15766/mep_2374-8265.11637

**Published:** 2026-09-17

**Authors:** Jennifer L. Vredeveld, Debra Burke, Rosalyn E. Maben-Feaster, Thomas S. Frederick, Michael J. Brenner, Allison L. Ruff

**Affiliations:** Associate Professor, Internal Medicine-Pediatrics, University of Michigan Medical School; Educational Services Specialist Lead, Quality – Education for Quality Improvement and Patient Safety, Michigan Medicine; Associate Professor, Obstetrics and Gynecology, University of Michigan Medical School; Continuous Improvement Specialist Lead, Quality – Continuous Improvement, Michigan Medicine; Associate Professor, Otolaryngology – Head & Neck Surgery, University of Michigan Medical School; Associate Professor, Internal Medicine, University of Michigan Medical School

**Keywords:** Quality Improvement, Process Mapping, Simulation, Process Optimization, Root Cause Analysis, Systems-Based Practice

## Abstract

**Introduction:**

Teaching quality improvement in medical education is challenging due to limited experiential learning. To address this, we implemented a patient care simulation utilizing building bricks to teach process mapping, root cause analysis, and iterative change.

**Methods:**

Second-year medical students (*N* = 480) participated in a 2-hour session simulating emergency department patient flow. Teams of 7-8 students assumed roles within the hospital, simulating steps in patient encounters, identifying problems, and testing iterative changes. Pre- and posttest assessments measured quality improvement knowledge and interprofessional competencies. Data collected from groups completing the simulation and annual postactivity reflections over 3 years were analyzed for themes.

**Results:**

Session quality ratings were significantly higher than other clinical-year health systems sessions in 2025 (4.23 vs 3.50), 2024 (4.15 vs 3.40), and 2023 (4.08 vs 3.51) on a 5-point Likert scale. In 2025, knowledge scores increased from 75.7% (*SD* = 1.13) pretest to 87.3% (*SD* = 0.894) posttest (*P* < .0002). By objective, gains were greatest for applying the scientific method to quality improvement, recognizing multiple root causes, and testing 1 countermeasure at a time (*P* < .001). Simulation data demonstrated sustained improvement in patient throughput.

**Discussion:**

This simulation engaged medical students and improved their understanding of health care systems, process optimization, and interprofessional teamwork. By engaging in root cause analysis and iterative improvement, students demonstrated enhanced competencies in quality improvement principles. Future iterations will refine the model and explore its application across additional clinical contexts.

## Educational Objectives

By the end of this activity, learners will be able to:

1. Apply the steps of the scientific method to quality improvement: Analyze a process map; Appraise data to develop change; Identify problems within a process; Design countermeasures to address identified obstacles to quality; Create and adjust SMART targets; Assess iterative experimentation as it applies to quality improvement; Compare differing approaches to quality improvement.
2. Describe how communication and team dynamics influence process improvement.
3. Analyze how understanding one's own role and the roles of others enhances team performance.
4. Evaluate how a single change can affect the broader system.

## Introduction

As health care systems grow more complex, medical students must be equipped not only to treat patients but also to diagnose and improve the systems in which they practice. Mastery of quality improvement (QI) principles—such as process mapping, data utilization, and implementation of countermeasures—is essential for improving health care outcomes and ensuring patient safety.^1^ However, opportunities for practical, hands-on experience are limited: clinical rotations emphasize disciplines rather than processes, and students rarely remain in 1 environment long enough to observe the impact of QI initiatives.^2,3^ Addressing this gap is critical, as early engagement with QI principles is foundational for future practice.^4,5^ Early exposure enables learners to recognize system inefficiencies, adopt a mindset of continuous improvement, and develop the skills necessary to implement sustainable change in clinical settings.^6^ Incorporating action-oriented QI education into medical curricula therefore supports professional development and strengthens health care systems.

Knowledge of QI principles—and the ability to apply them—is essential for success in medical school and on the USMLE, particularly as step 1 is often taken midway through training. Numerous national and international medical organizations, including the Accreditation Council for Graduate Medical Education (ACGME), the National Board of Medical Examiners (NBME), and the World Health Organization (WHO), have emphasized QI as a core component of medical education.^7-9^ The NBME supports this emphasis by offering undergraduate medical schools a dedicated health systems science (HSS) subject examination and by incorporating related content into the USMLE step 1 and step 2 examinations.^8^ Similarly, the Association of American Medical Colleges (AAMC) recently revised its patient safety and QI competencies, originally developed in 2019, further reinforcing the continued importance of this curriculum.^10^

As medical curricula continue to expand, educators face the challenge of incorporating QI and HSS content without further overburdening learners.^11^ To address this, QI education must be delivered in ways that are efficient, engaging, and closely connected to clinical practice. Gamification—the use of game design elements in nongame contexts, such as education—offers 1 approach to increasing learner engagement and motivation while making abstract systems concepts more concrete.^12^ This strategy may be particularly valuable for teaching topics that have been described as the “broccoli” of medical education: important and beneficial, but not always immediately appealing to learners.^11^ Prior work has demonstrated that gamified approaches can be successfully integrated into undergraduate medical education to enhance participation, reinforce key concepts, and support applied learning.^13-15^

Building bricks have long been used in educational interventions, with the Lego brand having a K-8 educational branch in its corporate structure.^16^ Building bricks have more recently been a welcome addition to hands-on education in university contexts.^16^ The use of play-based methods such as Lego Serious Play in higher education reflects constructivist learning principles and enriches students’ learning experiences by promoting active participation, metaphorical thinking, and reflection.^17,18^

Similarly, although gamification has previously been applied to QI education in a health care context, this intervention utilizing building bricks was designed to uniquely enable learners to observe the effects of small systems-level changes within an accelerated time frame. To our knowledge, this feature has not been demonstrated in other QI-based game strategies and fills a notable gap, as students are generally not present on the same rotation long enough to see the effects of systems changes. This session was developed as an interactive simulation to engage students in identifying and addressing health care system challenges using QI tools. With a simple building brick model, learners can visualize process flow, test small system changes, and observe their impact in real time. The activity provides early, practical exposure to QI principles in a low-risk, multidisciplinary team-based environment, helping students connect abstract concepts to tangible system behaviors while strengthening communication and collaboration skills.

## Methods

Second-year medical students, midway through their core clinical clerkships, participated in a week-long intensive focused on HSS, including principles of QI. As part of this program, we developed a 2-hour, hands-on simulation using building bricks, such as Legos, to model patient flow through a health care system. This immersive session gave students practical experience investigating problematic processes, collecting and analyzing data, and implementing iterative changes.

During the activity, students applied core QI tools such as process mapping, problem statement creation, and root cause analysis. They also completed multiple Plan-Do-Check-Act (PDCA) cycles to test and refine process changes in real time.

Students entered this simulation following a basic introduction to QI. Students had been introduced to the concept of QI in health care and the importance of understanding problems before suggesting solutions. Commonly used QI tools, including process mapping, root cause analysis, and iterative change, had been introduced but not previously practiced.

### Session Development

To make the medical school's QI curriculum more interactive and experiential, we developed this session through a collaboration between the medical school's QI curricular lead and the Department of Continuous Improvement. Building on the department's prior success with brick-based simulations and recognizing that students often have limited insight into how patients move through the health care system beyond their individual clinical roles, our team designed a simulation that uses building bricks to represent a patient's journey from emergency department (ED) arrival through hospital discharge.

Following the initial design, the session underwent multiple rounds of refinement. Colleagues participated in pilot sessions, providing feedback that informed iterative adjustments to both the simulation and game mechanics before implementation with students. The session was intentionally structured to promote critical thinking and problem solving with facilitator guidance rather than directing all teams through a single standardized approach. Since its launch, the simulation has continued to evolve through ongoing small improvements informed by annual student and facilitator feedback.

### Facilitators and Training

Each small-group session included 14-16 students, broken into 2 teams, and 2-4 trained facilitators. The facilitator team in each small-group room consisted of clinical faculty paired with QI specialists from the Department of Continuous Improvement to ensure that each skill set was represented in each room. Two weeks prior to the simulation, facilitators attended a training session that included a mock run-through of the activity and time for questions. We also provided a written facilitation guide to reference throughout the session (Appendix A). Curriculum leads were available throughout the actual simulation to provide real-time support and consultation.

### Materials

Materials needed for each small group (2 small groups in each room):

• 2 small bags that fit at least 20 building bricks for randomization
• 2 dice
• 42 blue bricks
• 44 red bricks
• 40 dark green bricks
• 20 yellow bricks
• 20 orange bricks
• 7 pink bricks
• Facilitation Guide (1 per facilitator, see Appendix A)
• Standard work for each role (printed double-sided on 8.5 x 11 paper, Appendix B)
• Hospital Game Board (24 x 36 paper/poster, laminated, Appendix C)
• 2 process maps (Print on 11 x 17, Appendix D)
• 2 RCA discussion guides (Appendix E)
• Data Collection Sheet (Appendix F)

Additional materials provided for groups to adjust the simulation as needed based on the countermeasures implemented:

• 2 dice
• 8 blue bricks
• 6 red bricks
• 5 dark green bricks
• 2 yellow bricks
• 2 orange bricks
• 3 pink bricks
• 20 lime green bricks

Additional materials provided for a bonus round if desired by students to test more patients coming into their ED:

• 20 dark green bricks for the ED nurse
• 10 yellow, 6 blue, and 8 red bricks for the ED physician
• 6 blue and 4 red bricks for the lab technician
• 10 red bricks for the transporter
• 10 orange bricks for the hospitalist

### Simulation Structure

Within each room, students split into 2 teams of 7-8 participants. Each student took on a role commonly found in the continuum of hospital care—from ED presentation to hospital discharge. Roles included triage nurse, ED nurse, ED physician, hospitalist, lab technician, hospital transport, environmental services, and QI officer. Some roles, such as hospital transport and QI officer, can be combined based on team size. We placed a printed copy of the standard work (see Appendix B) for each role face down around the Hospital Game Board (see Appendix C). Students were assigned their role randomly based on where they sat.

We designed the simulation to illustrate the complexity and interdependency of health care processes. The building bricks capture the patient's journey; a patient entering the system is a single building brick, and each step of that patient's care is marked by the addition of a colored brick by the provider responsible for each task. Red bricks denote high-acuity patients or abnormal labs necessitating hospital admission, whereas blue bricks represent low-acuity cases suitable for discharge. The hospital has 4 ED rooms and 3 inpatient rooms.

Patients are introduced into the system every 15 seconds via the triage nurse, who randomly selects a red or blue brick from a bag. The patient journey includes:

• Rooming by an ED nurse
• Evaluation by an ED physician
• Lab draw (ED nurse) and result reporting (lab technician)
• Reassessment by the ED physician
• Decision for discharge or hospital admission

Low-acuity patients discharged from the ED require room cleaning by environmental services—represented by rolling a die and achieving a result of 1, three times. High-acuity patients needing admission are transferred to the hospitalist, whose discharge process is also governed by die rolls—three 1s, then three 2s, then one 3 to represent rounding, meeting discharge criteria, and discharge. Patients cannot share rooms; every discharge requires room cleaning before reuse. High-acuity patients are prioritized and can displace lower-acuity patients. If no room is available, displaced patients are marked as having left against medical advice (AMA).

A properly treated building brick patient will display a specific brick sequence reflecting each care step. An image showing accurately discharged patients in this health care system is available in the QI officer's standard work and the Facilitation Guide. The QI officer audits each discharged patient's brick pattern to identify patient care errors and logs any AMA events using the Data Collection Sheet (see Appendix F). They also monitor the simulation in real time for errors, such as lack of room cleaning prior to reuse.

### Simulation Flow

The session began with students reviewing a brief role description. The QI officer demonstrated what a correctly processed patient should look like using the image on their standard work (see Appendix B). After a brief introduction, teams completed a 5-minute baseline simulation using standard work instructions based on their roles.

The QI officer collected and presented initial performance data using the Data Collection Sheet (see Appendix F), which students used to reflect on system inefficiencies. Teams analyzed a process map (see Appendix D) to deepen their understanding of individual roles and system flow. Improved communication and mutual understanding of the process constitute the first countermeasure implemented in the next simulation cycle.

Following the second simulation cycle and subsequent data review, teams constructed a problem statement and performed a root cause analysis with facilitator guidance using the RCA Discussion Guide (see Appendix E). Each team selected a root cause to address and designed a realistic countermeasure. The simulation is intentionally designed to allow critical and diverse thinking among student groups. Facilitators helped guide countermeasure discussions to avoid unrealistic solutions, such as building a new hospital wing or hiring additional staff. Only 1 countermeasure can be implemented each cycle. Modifications to the simulation were made to represent these interventions, and additional building brick pieces or dice may be used as needed.

Proposed countermeasures vary depending on the root cause identified by each team. For example, a team that identified delays in room cleaning may determine that environmental services is not consistently notified when a room becomes available. As a countermeasure, the team might implement an automated alert system to improve communication and reduce delays. Within the simulation, this change can be represented by modifying the die rolls or adding an additional die to reflect improved efficiency.

Alternatively, teams may observe that a large number of patients are admitted from the ED, including some whose abnormal laboratory results could be managed safely as outpatients. In response, they may propose a process to expedite outpatient follow-up for selected abnormal lab findings, allowing more patients to be discharged directly from the ED. This change can be incorporated into the simulation by adding a new color brick for the lab technician to represent patients with abnormal labs who are eligible for outpatient management.

The primary goal of this exercise is to encourage critical thinking, creativity, and systems-based problem solving. With facilitator guidance, students developed and tested interventions while monitoring the system for both intended and unintended consequences of change.

Teams set measurable improvement goals before running 1-2 additional simulation cycles, incorporating data analysis and iterative refinement after each.

Teams that met or exceeded their goals may have been challenged with increased patient volume to test the durability and scalability of their improvements.

The session ended with a group debrief, where each team shared their observations, challenges, and outcomes. Facilitators led a discussion to help students connect the simulation to real-world clinical systems and reflect on the application of QI tools and methodologies in practice.

### Data Analysis

We designed an assessment (Appendix G) to measure QI knowledge based upon learning objectives as well as confidence in QI skills. We administered this assessment both before and after the session to collect pre- and posttest data. The course director and content lead, both of whom have significant educational and QI backgrounds, designed assessments for internal program evaluation rather than research validity, and therefore items were not previously validated. We graded knowledge questions utilizing binary scoring. Confidence questions were assessed pre- and postactivity utilizing a 5-point Likert scale with a score of 1 being not at all confident and a score of 5 correlating to extremely confident. Pre- and postknowledge test data was analyzed using a Wilcoxon signed-rank test analysis. Confidence data was analyzed using a Bonferroni correction, requiring a *P* value less than .0055 to demonstrate significance. Data collected by teams during the 2025 session were reviewed to assess for system improvements. Real-time feedback was collected at the end of each session over the 3-year period using sticky notes as an internal program evaluation tool. Students were asked to record 1 “Aha moment” (something they learned during the session) and 1 piece of feedback on provided sticky notes and post them on a designated wall as they exited. Postactivity reflections over 3 years were qualitatively analyzed by program leadership to identify themes.

This study was reviewed by the University of Michigan Institutional Review Board (HUM00270889) and determined to be exempt from Institutional Review Board oversight under Exemption 1 at 45 CFR 46.104(d)(1), pertaining to research conducted in established or commonly accepted educational settings involving normal educational practices.

## Results

This session was repeated annually over 3 years, engaging a total of 480 medical students. Session quality scores as noted by the students, which are obtained universally for all student-facing sessions in the curriculum, were 4.23 (2025), 4.15 (2024), and 4.08 (2023) on a Likert scale of 1-5, significantly higher than the average of 3.50 (2025) 3.40 (2024), and 3.51 (2023) for other health systems educational sessions in the clinical year. In 2025, pre- and posttests were given to assess the learning objectives noted previously. Survey response rates were 77% (125/163) presession and 39% (63/163) postsession. Pretest scores averaged 75.7% (*SD* = 1.13), improving to 87.3% (*SD* = 0.894) postsession (*P* < .0002). Of note, not all respondents answered every survey question. Breaking the scores down by learning objectives, the simulation was especially noted to be beneficial in learners’ ability to apply steps of the scientific method in a QI setting, understanding that most problems in health care stem from more than 1 root cause, but only 1 countermeasure should be applied at a time (*P* < .001). Other areas were more challenging to demonstrate statistically significant improvement, potentially due to high pretest scores and low posttest response rate (Table 1).

**Table 1.**
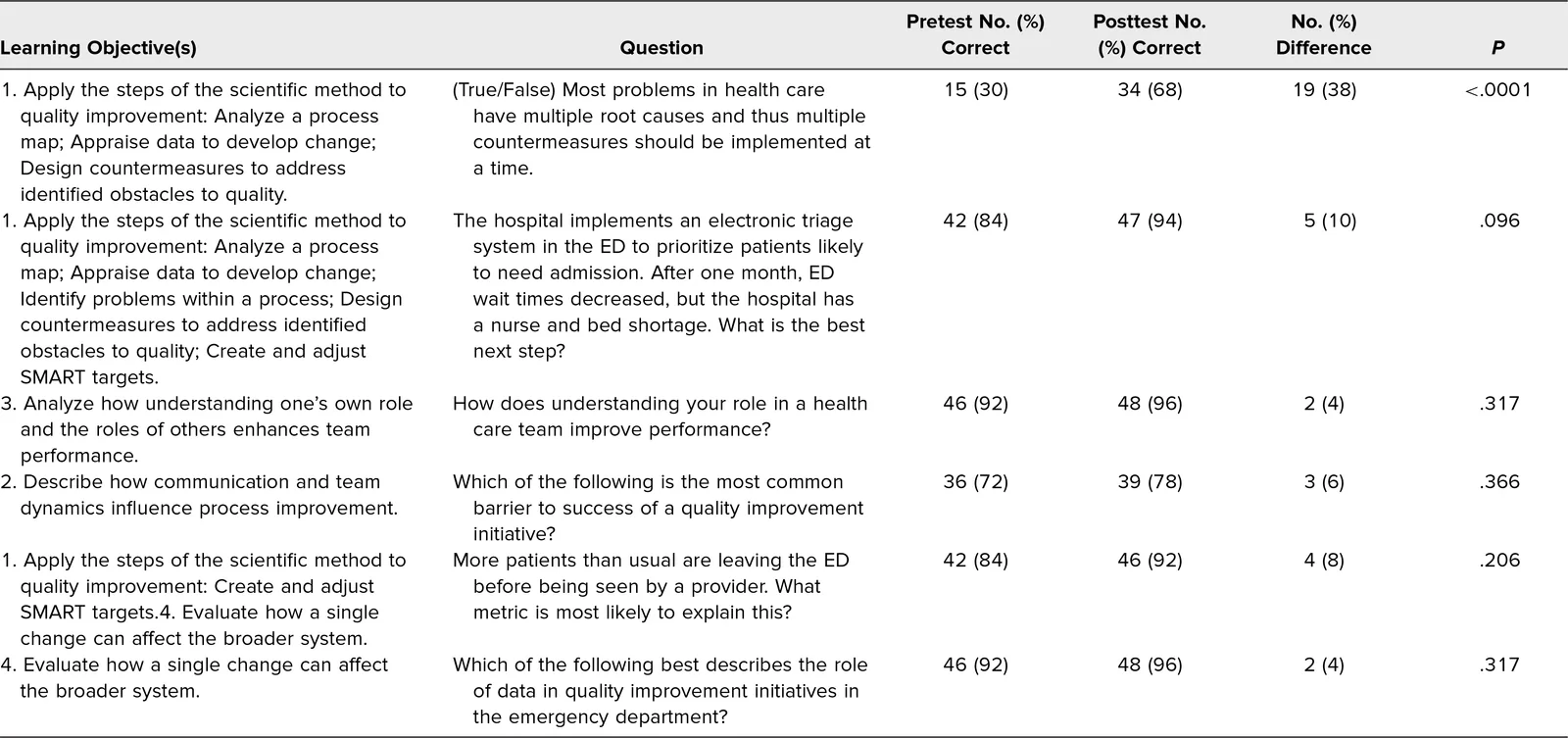
Test Responses to Questions That Address the Learning Objectives by Session Participants Who Completed Both Pre- and Postsurveys (*N* = 50)

In the same pre- and postsession surveys, students also noted improved confidence in performing QI tasks, including understanding the roles of other health professionals in a process, using data in QI, process mapping, creating a well-defined problem statement, performing a root cause analysis, identifying countermeasures to address root causes, assessing the impact of change in QI, and identifying SMART (specific, measurable, achievable, relevant, and time-bound) goals. In all cases, a *P* value less than .0001 demonstrated a statistically significant change in learner confidence (Table 2).

**Table 2.**
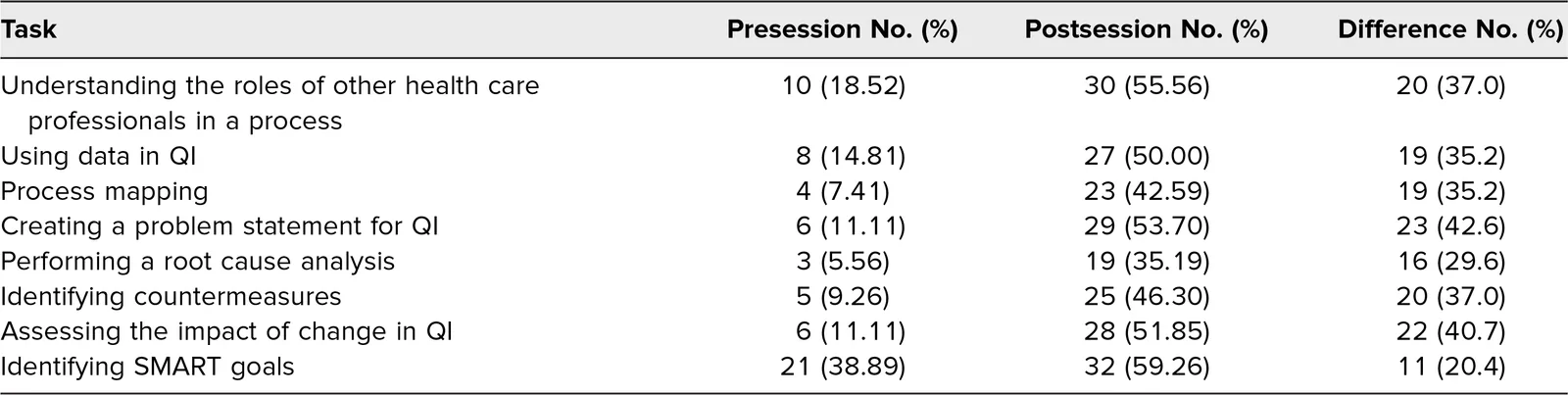
Session Confidence Ratings as Very Confident or Extremely Confident in Quality Improvement-Related Tasks for Students Completing Both Pre- and Postsurveys (*N* = 54)

Following the 2025 session, data collection sheets from 19 of 20 groups were reviewed (1 group failed to return their sheet). Patient throughput improved consistently across all groups. Total successful discharges increased from 104 during the baseline cycle to 347 by the third iterative change. Concurrently, both AMA discharges and process-related errors declined. AMA discharges decreased from 119 at baseline to 44 after the third cycle. Similarly, total documented errors (incorrect brick pattern simulating patient care error or lack of room cleaning between patients) fell from 141 to 21 across the same period. Notably, 11 of the remaining errors in the final cycle originated from a single group that had reported zero errors initially. The Figure summarizes the composite data from all 19 groups reviewed.

**Figure.**
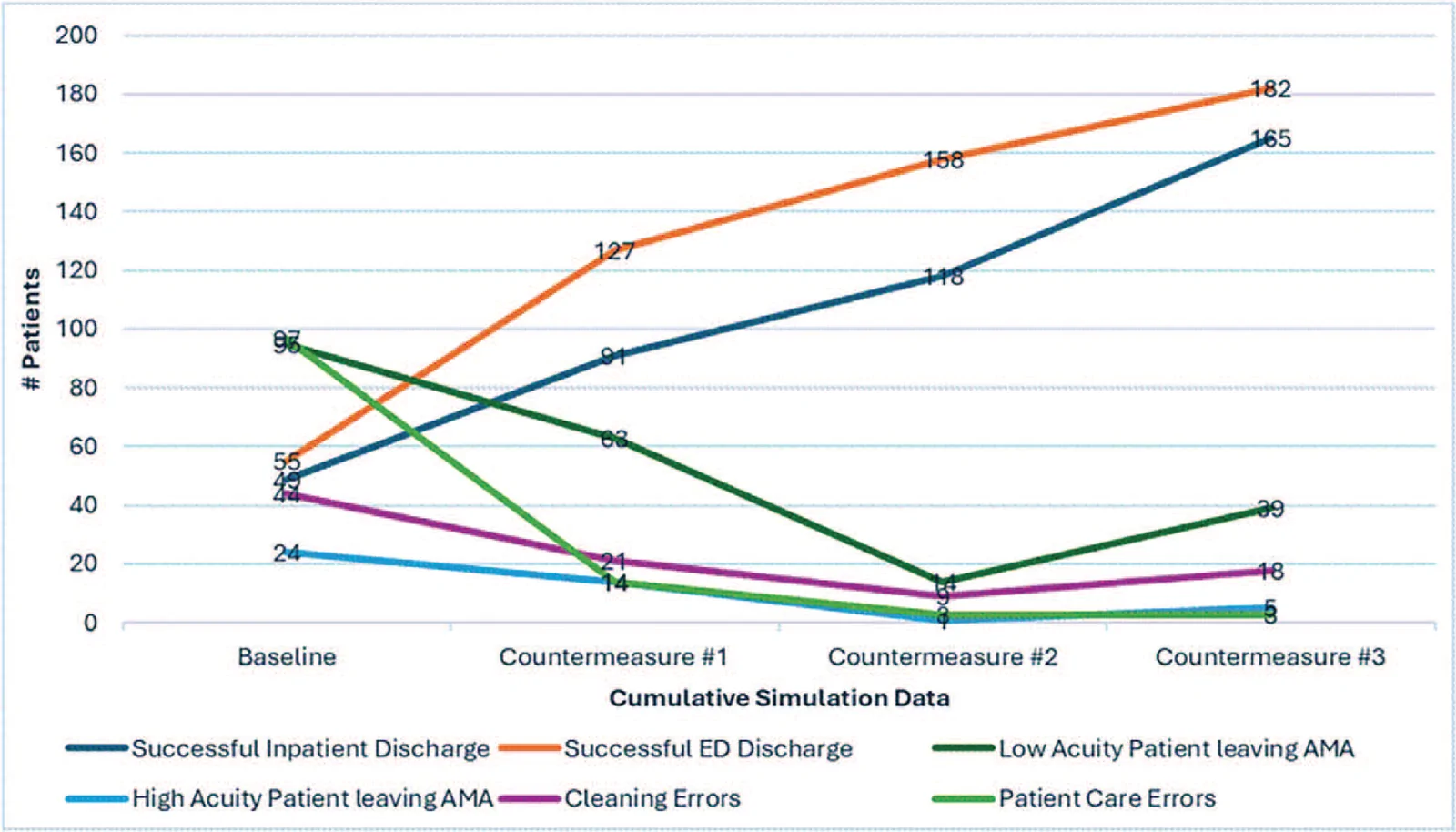
Cumulative simulation data from all groups participating in the 2025 session showing accurate patient throughput (successful inpatient and ED discharges), AMA discharges, and system errors (incorrect patient care and/or cleaning errors). Abbreviations: AMA, against medical advice; ED, emergency department.

Based on the free response feedback collected on sticky notes at the end of each session, we identified several recurring themes. Students most commonly emphasized communication and teamwork (“Communication is everything!” and “Defined roles make the system work smoothly”). They also frequently noted an awareness of systems thinking, including recognition that “bottlenecks shift around when changes are implemented” and that “solutions can create new problems.” Additionally, students described greater understanding of QI principles, particularly process mapping and root cause analysis (“You need to ask ‘why’ more than expected” and “RCA is effective in identifying problem areas and then starting to find the most effective solutions”). Finally, students valued the hands-on format and clinical relevance, describing the session as “super engaging and fun,” a “great opportunity to practice root cause analysis,” and helpful in developing “a much better understanding of the workflow of the hospital.”

## Discussion

This novel small-group session demonstrates that an interactive, simulation-based session using building brick models can significantly enhance medical students’ understanding of and confidence in applying key QI principles. These findings support the literature suggesting that active, hands-on QI training promotes deeper comprehension and skill development than lecture-based instruction alone.^19,20^ This also adds to previous literature demonstrating the use of building bricks for learning in higher education.^21,22^

The improvement in learners’ posttest performance, particularly in applying the scientific method to QI and understanding the importance of limiting countermeasures to 1 change at a time, underscores the effectiveness of experiential, iterative learning. Simulation data showing significant improvement in efficiency and patient throughput while simultaneously decreasing errors further demonstrates students’ ability to use QI tools to make systems improvements. These gains are consistent with principles of adult learning theory and Bloom's taxonomy, which emphasize application, analysis, and evaluation as critical steps in achieving higher-order learning.^23^ By allowing students to directly manipulate a simulated health care system, test interventions, and observe outcomes in real time, this curriculum bridges the gap between abstract QI theory and practical implementation.

Student reflections reinforced the quantitative results, with recurrent qualitative themes highlighting the importance of communication, teamwork, and systems thinking. These insights reflect the fundamental competencies outlined in the ACGME domains of Practice-Based Learning and Improvement and Systems-Based Practice.^7^ Learners recognized the interdependent nature of health care processes and the impact of small system changes on broader outcomes, suggesting that the simulation effectively fostered a mindset of continuous improvement and systems awareness. Additionally, the positive affective responses—describing the exercise as “fun,” “engaging,” and “realistic”—indicate that experiential simulations can enhance motivation and retention.

Unexpectedly, qualitative feedback also focused on siloing, a concept not directly addressed in the learning objectives. Students noted that physicians are shielded or siloed away from many of the challenges of process improvement, including the roles of others on the care team with whom they do not typically interact—environmental services and hospital transport. This provides an important interprofessional perspective that is more commonly covered in interprofessional education curricula.

While the assessment-based data is limited due to high pretest scores, low posttest response rate, and imperfect assessment alignment with learning objectives, students reported significant improvements in confidence related to performing tasks/skills noted in the learning objectives. Change in self-reported confidence aligns with level 2 in the New World Kirkpatrick model and is evidence of efficaciousness of the intervention.^24^ Additionally, the consistently high session ratings across 3 academic years further validate the session's reproducibility and value. The alignment of facilitator expertise in both clinical practice and QI methodology likely contributed to the session's success by modeling interdisciplinary collaboration and reinforcing practical application. Moreover, the low-cost, scalable nature of the building brick-based simulation supports its feasibility for adoption across diverse educational settings, making it accessible to institutions seeking to strengthen QI education without significant resource demands.

Despite these strengths, several limitations should be acknowledged. The evaluation relied on self-reported confidence measures and short-term knowledge assessments, which may not reflect long-term retention or translation into clinical practice. Furthermore, evaluation and feedback were completed initially through student surveys, with assessment of knowledge acquisition evaluated only in the most recent interaction. Knowledge questions were developed for programmatic evaluation, did not equally assess all learning objectives, and thus were not vetted for research purposes. Additionally, only 63 students completed both the pre- and postactivity surveys, and students who opted to complete these may have had a different reflection on their learning than those who did not.

Future iterations should consider using a validated instrument for assessing QI knowledge as well as examine whether participation in such simulations influences students’ subsequent engagement in QI projects or their ability to lead system-level improvements during residency and beyond.

While a building brick-based simulation activity may not fully replicate real-world constraints, workflow variability, or human factors, it offers a flexible and engaging hands-on approach to teaching QI concepts that can be integrated into a variety of HSS curricula. This scalable approach can help medical students develop the systems-thinking skills and improvement mindset that underpin high-quality patient care.

## Appendices


Facilitation Guide.docxStandard Work.docxHospital Game Board.pptxProcess Map.pptxRCA Discussion Guide.docxData Collection Sheet.docxSession Assessment Questions.docx
*All appendices are peer reviewed as integral parts of the Original Publication.*
