# Supplementary material for: From Pieces to Processes: Teaching Health Care Process Optimization and Quality Improvement Through a Building Brick Simulation
Source: MedEdPORTAL. 2026 Sep 17;22:11637. doi: 10.15766/mep_2374-8265.11637 (PMC13582539; doi:10.15766/mep_2374-8265.11637)
Supplement: Supplementary file 1 [file mep_2374-8265.11637-s001.zip › E. RCA Discussion Guide.docx]

**Guide for White Board Discussion**

**Problem Statement**: Review the data collection sheet and the process map. What is the area you want to focus on to improve?

**Root Cause Tree**

State the Problem here…

Cause #3

Cause #1

Cause #2

**Counter Measure**: Based on the root cause tree, what are some possible

options to try to improve the problem?

Why?

Why?

Why?

Why?

Why?

Why?

Why?

Why?
