# Supplementary material for: From Pieces to Processes: Teaching Health Care Process Optimization and Quality Improvement Through a Building Brick Simulation
Source: MedEdPORTAL. 2026 Sep 17;22:11637. doi: 10.15766/mep_2374-8265.11637 (PMC13582539; doi:10.15766/mep_2374-8265.11637)
Supplement: Supplementary file 1 [file mep_2374-8265.11637-s001.zip › F. Data Collection Sheet.docx]

| Cycle | Discharges | | AMA Discharges  (Against Medical Advice/Left without being seen) | | Errors | | Boarding  (Waiting in the ED for an Inpatient Bed) |
| --- | --- | --- | --- | --- | --- | --- | --- |
|  | Inpatient | ED | Low Acuity | High Acuity | Cleaning | Patient Care  (incorrect brick pattern) |  |
| BASELINE |  |  |  |  |  |  |  |
| COUNTERMEASURE #1  Process Map and Understanding Roles  Improvement Goal: |  |  |  |  |  |  |  |
| COUNTERMEASURE #2  Improvement Goal: |  |  |  |  |  |  |  |
| COUNTERMEASURE #3  Improvement Goal: |  |  |  |  |  |  |  |
