# Supplementary material for: From Pieces to Processes: Teaching Health Care Process Optimization and Quality Improvement Through a Building Brick Simulation
Source: MedEdPORTAL. 2026 Sep 17;22:11637. doi: 10.15766/mep_2374-8265.11637 (PMC13582539; doi:10.15766/mep_2374-8265.11637)
Supplement: Supplementary file 1 [file mep_2374-8265.11637-s001.zip › G. Session Assessment Questions.docx]

**Pre/Post Assessment Questions**

1. True or **False**: Most problems in healthcare have multiple root causes and thus multiple countermeasures should be implemented at a time.
2. The hospital implements an electronic triage system in the ED to prioritize patients likely to need admission. After one month, ED wait times decreased, but the hospital has a nurse and bed shortage. What is the best next step?
a) Discontinue the triage system and return to the previous workflow.
b) **Investigate the impact of the triage system on inpatient workload and adjust the admission process accordingly.**
c) Require inpatient teams to adjust their staffing to accommodate increased volume.
d) Collect data for another month, to get further insights since the problem might sort itself out.
3. How does understanding your role in a healthcare team improve performance?
   a) It prevents unnecessary work
    b) It helps ensure all team members stay within their job descriptions
    **c) It improves coordination and reduces misunderstandings**
    d) It allows individuals to work independently without distractions
4. Which of the following is the most common barrier to success of a quality improvement initiative?
a) Low employee morale
b) **Lack of clear communication**
c) Excessive training/expertise
d) Resource utilization
5. More patients than usual are leaving the ED before being seen by a provider. What metric is most likely to explain this?
a) Patient satisfaction scores
b) **Time from check in to seen by a provider**
c) Provider satisfaction scores
d) Percentage of patients admitted to the hospital
6. Which of the following best describes the role of data in quality improvement (QI) initiatives in the emergency department (ED)?
a) Data should be collected only after a process change has been implemented to determine its effectiveness.
b) Data is primarily used to identify individuals responsible for system failures.
c) **Data should guide decision-making, helping to identify bottlenecks and direct change through process mapping and countermeasures.**
d) Data collection is unnecessary if the problem is already well understood
7. Please rate your current level of confidence in performing the following quality improvement (QI) tasks:

|  | Not at all confident | Slightly confident | Somewhat confident | Very confident | Extremely confident |
| --- | --- | --- | --- | --- | --- |
| Understanding the roles of other healthcare professionals in a process |  |  |  |  |  |
| Using data in quality improvement |  |  |  |  |  |
| Process mapping |  |  |  |  |  |
| Creating a problem statement for QI |  |  |  |  |  |
| Performing a root cause analysis |  |  |  |  |  |
| Identifying countermeasures |  |  |  |  |  |
| Assessing the impact of change in QI |  |  |  |  |  |
| Identifying SMART goals |  |  |  |  |  |
