# Supplementary material for: From Pieces to Processes: Teaching Health Care Process Optimization and Quality Improvement Through a Building Brick Simulation
Source: MedEdPORTAL. 2026 Sep 17;22:11637. doi: 10.15766/mep_2374-8265.11637 (PMC13582539; doi:10.15766/mep_2374-8265.11637)
Supplement: Supplementary file 1 [file mep_2374-8265.11637-s001.zip › A. Facilitation Guide.docx]

**Session Objectives:**

Use a hands-on, interactive, small group simulation exercise to:

- Practice using the scientific method
  - Use a process map to better understand a process
  - Use data to understand, direct and evaluate change
  - Identify obstacles to quality and countermeasures to address them
  - Implement countermeasures and measure their effectiveness
  - Create and adjust SMART targets
  - Practice iterative experimentation as it applies to quality improvement
  - Track improvement efforts over time
  - Reflect on how quality improvement efforts must be balanced with customer value
- Describe how communication and team dynamics can improve processes
  - Analyze how understanding one’s own role and the roles of others enhances team performance
- Evaluate how isolated change impacts the larger system

**Facilitator Review:**

- The activity is set up to have 2 teams of 7-8 students per room with 2-4 facilitators.
- When possible, 1 of the facilitators will have a clinical background. This is to help the students with any clinical questions they may have regarding the scenario. Other facilitators will have a background in quality improvement.
- Facilitators should use their discretion regarding timing and learning objectives to determine the amount of discussion required for ANY questions. The topic can be added to a “Parking Lot” to be discussed later if time allows.
- Keep the two teams in the room on the same schedule. It will be very difficult to facilitate the teams individually, it can get very noisy. If you need to wait for the other team to catch up that is ok. Make sure you are communicating with the other facilitators to keep this on track.
- Determine the assignments for the facilitators in the room. (each facilitator will have multiple roles)
  - Time Keeper
  - Main script reader
  - 1 facilitator per table/team during each simulation and to guide the smaller group reflections
  - anything else as needed
- The simulation will be set up prior to the session by the faculty leads. Please arrive early to make sure all materials are set in the room and facilitators in your room know their roles.
- This session is for 2nd year medical school students.

| Example Times | Length of time | Task | Script | Tips |
| --- | --- | --- | --- | --- |
| Pre session |  | Set up Simulation |  |  |
|  |  | Kit Contents:  2 small bags for triage nurse and lab tech. (Building bricks will be put inside so that selection can be randomized)  2 dice (+ 1 or 2 extra)  42 Blue Building bricks (50 in kit, 8 extra)  44 Red Building bricks (50 in kit, 6 extra)  40 Dark Green Building bricks (45 in kit, 5 extra)  20 Yellow Building bricks (22 in kit, 2 extra)  20 Orange (22 in kit, 2 extra)  7 Pink Building bricks (10 in kit, 3 extra)  20 Lime Building bricks  Painters tape  Bonus Round Kit if needed  (6 Blue, 4 Red)  Please document any feedback for the session on this facilitation guide. Please focus attention on:   1. Where Sim needs points of clarification 2. Timing adjustments   Overall Schedule (For Reference)   - Introductions - Learning Rules - Run Simulation #1, collect data - Debrief (Process Map) - Run Simulation #2, collect data - Debrief (Problem Statement, RCA) - Determine Change - Run Simulation #3, collect data - Debrief - Determine Change - Run Simulation #4 (if time), collect data - Final Debrief and share out between teams regarding experience and lessons learned. | Ask students not to flip over their papers until told to do so. | Set up guidelines (Refer to last page for picture of set up)  Triage Nurse: 14 Blue, 6 Red (in bag)  ED Nurse: 40 Dark Green  ED Physician: 20 Yellow, 14 Blue, 12 Red  Lab Technician: 14 Blue, 6 Red (in bag)  Transport: 20 Red  Environmental Services: 7 Pink (to be placed by rooms) and 1 dice  Hospitalist: 20 Orange and 1 dice  QI Officer: Data collection sheet  Use painters tape to adhere the Game Board (ED rooms and inpatient rooms) to table  20 Lime Green have been added to the kit for optional use as a countermeasure. These would be given to students after the first baseline cycle if requested or the facilitator may identify if it would aid in the countermeasure implementation.  Some teams may have time or want an extra challenge. A bonus round kit has been included that can be added to the base game during round 4.  **Building bricks for Bonus Round (if needed)**  Triage Nurse: add 6 Blue, 4 Red to bag  ED Nurse: add 20 Dark Green  ED Physician: add 10 Yellow, 6 Blue, 8 Red  Lab Technician: add 6 Blue, 4 Red  Transport: add 10 Red  Environmental Services: No additions required  Hospitalist: add 10 Orange  QI Officer: No additions required  (extra Building bricks are for backup in case any become missing) |
| 10:00-10:05 | 5 | Introduction |  |  |
|  |  | Introduce yourself.  Have Students Introduce themselves - Names only, keep it quick  Introduce the day | “This session is to provide a hands-on, interactive, small group simulation exercise to practice using the scientific problem-solving method. We will have about 2 hours and run the simulation 4x. ”  “Who is the ED Nurse? The ED Nurse is the most complicated role. If you don’t want to take this on, that is ok, please feel free to switch with someone who would like that role.”  “Additionally, if anyone identifies something that will prevent them from participating fully, such as being able to determine colors of the Building bricks, or is restricted by any movements that may be required, once we have turned over the instruction sheets, please bring that to our attention and we will help work that out.”  “Go ahead and turn over the pages” | Some of the students may not know each other.  **If there are less than 8 students the transport/QI officer roles can be combined**  If there are less than 7 students per team:   - If 2nd team in room has 8, please redistribute students - If you need to get started before 7th student arrives please have one of the facilitators take a role so activity can get started |
| 10:05-10:10 | 5 | Learn simulation role |  |  |
|  |  | Direct students to the standard work at their seat | “Go ahead and read the standard work of the role you have in front of you. Become familiar with what your task will be”  “Before we get started I will give you a couple of hints. QI Officer - can you please show your team the image of what correctly discharged patients look like?”  “As you can see, as you complete the task assigned to your role you will be stacking or locking your building bricks on top of the previous task”  “I hope that helps, let's get started” | Students may have questions about their individual roles. Answer these at your own discretion, but do not allow questions to delay the start of cycle 1. They will have time after cycle 1 to better learn their roles.  For reference, a photo of correctly discharged patients is on the QI officer’s standard work and looks like the following:    (Author Owned Photo)  **Keep this part to 5 min or less. If there is a lot of table discussion go ahead and move to the activity.** |
| 10:10-10:15 | 5 | SIMULATION Round 1 |  |  |
|  |  | Have one facilitator start the simulation. Give a 1 min warning and then stop the simulation at 5 minutes. | “You have 5 min, Go” | During the simulation, the students will be uncomfortable with things that they do not understand. If the team is starting to be stressed about the scenario, providing them with some guidance is appropriate. However, refrain from giving them a lot of instruction. It is expected that the first round will be somewhat chaotic as they only know their own role and have little understanding of how the system functions. |
| 10:15-10:20 | 5 | Data Collection |  |  |
|  |  | Ask the QI officer to complete data collection.  Ask students to reset the simulation once the data collection role has tabulated all data. | “Pause and observe the state of the house, you can take an actual picture to reflect on later if you want”  “The QI officer has a data collection sheet to collect your data”  “Once the data is collected, everyone else please reset the simulation to run the next round. There is guidance on each of your role descriptions” | The group may decide on additional metrics to record. If needed, facilitators can help guide them through this. |
| 10:20-10:30 | 10 | Process Map/Reflection/Small Group Learning |  |  |
|  |  | Each of the small groups should do their own debrief. **Facilitators will need to help students with their discussions.**  Hand out 2 copies of the process map. The blue tape can be used to adhere 1 of the copies to the wall or the discussion can be at the table.  They will not be making an additional change in this round. The first intervention is understanding process and roles. It should show students the impact of understanding a process and the roles of other healthcare professionals. We will continue to emphasize the importance of making only 1 change at a time. | “Did anyone find that a little chaotic?”  “Why/Why not?”  “One of the first steps in understanding problems is learning about the process related to those problems, what our role is within that process, and the perspectives of our colleagues who are also involved in the process.”  **“Take a few minutes to walk through the process at this hospital using the process map provided. Feel free to use that map to take notes or signal areas of confusion/chaos.”** | Have each student share their role and standard work as they go through the process map. This will simulate them going and learning about what other roles are responsible for.  Encourage them to identify problematic steps in the process as they go through the mapping process. |
| 10:30-10:35 | 5 | SIMULATION Round 2 |  |  |
|  |  | Have one facilitator start the simulation. Give a 1 min warning and then stop the simulation at 5 minutes. |  |  |
| 10:35-10:40 | 5 | Data Collection |  |  |
|  |  | Ask the QI officer to complete data collection.  Ask students to reset the simulation once the data collection role has tabulated all data. | “Pause and observe the state of the house, you can take an actual picture to reflect on later if you want”  “The QI officer has a data collection sheet to collect your data”  “Once the data is collected, everyone else please reset the simulation to run the next round. There is guidance on each of your role descriptions” | The group may decide on additional metrics to record. If needed, facilitators can help guide them through this. |
| 10:40-10:55 | 15 | Debrief/Reflection/Small Group Learning- RCA |  |  |
|  |  | Each of the small groups should do their own debrief. Facilitators will likely need to help students with their discussions.  Pass out 2 “Guide for White Board Discussion'' per team (students will share) The blue tape can be used to adhere the 1 of the copies to the wall or have the discussion at the table.  1. Have students use the process map to identify areas of chaos, waste, wait time, poor quality, etc.  2. Identify 1 of the areas as a problem. (Develop a problem statement)  3. Perform a root cause analysis with 5-why, fishbone, RCA tree or a discussion.  4. Decide on one countermeasure to implement. Document this on the data collection sheet.  5. Set improvement goal. Document this on the data collection sheet. | “What was done well? What needs improvement/What are potential problem areas?”  “Compare data from this cycle to the first”  Referring to the “Guide for white board Discussion”, ask students to complete each of these tasks. A few reminders to give students:   1. Be careful when creating a problem statement as many people tend to create statements that are actually solutions in disguise 2. Consider using your data to help you identify a problem to improve 3. A strong problem statement identifies a gap to close 4. When considering root causes, try to expand your thinking beyond what seems to be obvious (like you would when you’re creating a differential diagnosis) 5. Countermeasures should be realistic- we can’t simply hire more staff or build additional rooms. After considering a realistic countermeasure, use creative ways to translate that into a change for the simulation. 6. Only 1 countermeasure should be implemented at a time. | We have 15 total minutes for this work. The expectation is that you will guide the students through the problem statement, root cause tree, and countermeasures. The students will have a guide for whiteboard discussion to look at. The facilitator will be up at the whiteboard leading the discussion.  Please look at each group’s problem statement. **Students have a tendency to create problem statements that are solutions in disguise like “We don’t have enough housekeepers.” Encourage them to look at their data. If needed ask some prompting questions** to help them:   1. What do you think about the proportion of the patients that come to your ED and leave AMA? 2. Do you find the number of patients boarding in your ED to be acceptable? 3. How many high acuity patients left without being seen? What should that number be? 4. Did you have any errors? Is that an acceptable number of errors?   Please provide guidance as needed. They do not need to develop a perfect problem statement. We are limited on time.  Guide students through a root cause tree, helping them focus on one branch to get them to a root cause.  When thinking about countermeasures, encourage students to think of realistic changes. They will likely want to change the die rolling for Environmental Services or the hospitalist. Only let them do this if they can articulate a realistic way in which those processes may become more efficient. Examples:   1. Cleaning rooms after patients who had c.diff requires 6 hours, but they decide to change the process of cleaning (a new protocol/technique). This may make Environmental Services more efficient and could result in them getting an additional dice. 2. They develop a better way to signal Environmental Services on the need for room cleaning that decreases the delay from time of discharge to the beginning of cleaning. This makes the process more efficient. 3. Prolonged admissions- implementing changes to rounding, the discharge process, etc may result in the hospitalist changing their rolling requirements or getting an additional die. 4. Should all high acuity patients be admitted? Is there a way to decrease the need for admission with rapid outpatient follow up or better anticipatory guidance? If so, they could identify a portion of these patients that are acceptable for discharge from the ED. 5. Is there benefit in having the lab wait for 2 samples before running a test? Could elimination of batching improve patient flow?   There are also additional lime green building bricks provided if there is a countermeasure they identify where these could be helpful (a change in an alert system or alteration of task management by different individuals on the team. Though remember that this should still be realistic… they should not ask the transporter to draw blood in the ED)  Discourage students from implementing multiple countermeasures at once (this will likely come up in several groups)  Allow students to be creative with their countermeasures and manipulation of the system, but discuss the practicality of their proposed changes outside of this simulation and refrain from allowing changes centered around additional resources or cost. |
| 10:55-11:00 | 5 | Modify simulation |  |  |
|  |  | Have students set up simulation with identified countermeasure. | Ask each group to set goals for their metrics based on their analysis of the baseline data and perceived impact of their proposed countermeasure. | There is space on the QI office data collection sheet to record this. |
| 11:00-11:05 | 5 | Simulation Round 3 |  |  |
|  |  | Have one facilitator start the simulation. Give a 1 min warning and then stop the simulation at 5 minutes. |  |  |
| 11:05-11:10 | 5 | Data Collection |  |  |
|  |  | Ask the QI officer to complete data collection.  Ask students to reset the simulation once the data collection role has tabulated all data. | “Pause and observe the state of the house, you can take an actual picture to reflect on later if you want”  “The QI officer has a data collection sheet to collect your data”  “Once the data is collected, everyone else please reset the simulation to run the next round. There is guidance on each of your role descriptions” | The group may decide on additional metrics to record. If needed, facilitators can help guide them through this |
| 11:10-11:25 | 15 | Debrief/Reflection/Small Group Learning |  |  |
|  |  | Each group should do their own debrief (they will share their experiences at the end)   1. Compare data from prior cycles. Review results compared to improvement goal set 2. Refer back to the Root Cause Analysis and adjust or expand if needed 3. Identify another countermeasure to implement  - This can be a different countermeasure to address the same root cause or a different root cause  4. Document the planned countermeasure and improvement goal on the data collection sheet | “How did this round go? What needs improvement/what were potential problem areas?”  “Either revisit your current problem statement and RCA or develop a new problem statement and RCA.”  “Decide on a countermeasure and set an improvement goal. Record your improvement goal.”  “Make sure you give yourself enough time to set up the simulation with the new countermeasure.” | After goal is identified have students set up simulation with modified countermeasure.  Remind teams that they will make only one change. Talk about how to prioritize, what will have greatest impact on the goal they want to change  What may only have a moderate impact, but is a very simple/easy to implement change?  Discourage changes that require the addition of resources/money. However, they can repurpose current resources if realistic (such as making the ED hallway space an acceptable place to triage certain low acuity patients to be seen)  **Some groups may have made significant improvements during their 2nd and 3rd simulations. If this is the case, consider challenging them to sustain these changes while adjusting for changing conditions in the ED by having patients arrive every 10 seconds rather than every 15 seconds. You will need to pass out the extra materials in the bonus round bag. Have each role add the following building bricks to their base kit.**  **Building bricks for Bonus Round (if needed)**  Triage Nurse add 6 Blue, 4 Red to bag  ED Nurse add 20 Dark Green  ED Physician add 10 Yellow, 6 Blue, 8 Red  Lab Technician add 6 Blue, 4 Red  Transport add 10 Red  Environmental Services No additions required  Hospitalist add 10 Orange  QI Officer No additions required |
| 11:25-11:30 | 5 | Simulation round 4 |  |  |
|  |  | Have one facilitator start the simulation. Give a 1 min warning and then stop the simulation at 5 minutes. |  |  |
| 11:30-11:35 | 5 | Data Collection |  |  |
|  |  | Ask the QI officer to complete data collection.  Ask students to reset the simulation once the data collection role has tabulated all data. | “Pause and observe the state of the house, you can take an actual picture to reflect on later if you want”  “The QI officer has a data collection sheet to collect your data” | The group may decide on additional metrics to record. If needed, facilitators can help guide them through this |
| 11:35-11:40 | 5 | Small Group Debrief |  |  |
|  |  | Reflect and compare data from all cycles  Review results compared to improvement goal set | “Did you sustain your change from cycle 1?” |  |
| 11:40-11:50 | 10 | Large Group Debrief |  |  |
|  |  | Have each group share their findings with the other   1. Problem statement 2. Problem areas identified during process mapping 3. Root causes identified 4. Countermeasures implemented 5. An overview of their data 6. Future steps considered   **Have students write on a sticky note their overall takeaway PLUS any other feedback.**  If you have a few minutes left you can ask a few students to share their thoughts about the session. | “One last thing before you leave, if you could write a takeaway or a-ha moment PLUS any other feedback on the sticky notes.”  Please leave this feedback on the wall by the door as you leave. | **QI officer can be the default presenter if the team is struggling with this.**  Place recommended points to share on white board to guide the presenter   1. Problem statement 2. Problem areas identified during process mapping 3. Root causes identified 4. Countermeasures implemented 5. An overview of their data 6. Future steps considered   Each group will only have about 5 min. Keep an eye on time.  Engage groups in discussion as they report. Use their findings to insert teaching points where appropriate. As an example, sometimes as a team's efficiency improves, their errors increase. Use opportunities like this to discuss things like balancing measures and how this may be considered and monitored in a real project.  **Make sure to celebrate each team at end of presentation.**  Have students place the sticky notes with takeaway and feedback by door as they leave. |

**Visual of how the activity set up could look. Ensure each role has the materials identified on the sheet at that position**

(Author Owned Photo)
