# Supplementary material for: From Pieces to Processes: Teaching Health Care Process Optimization and Quality Improvement Through a Building Brick Simulation
Source: MedEdPORTAL. 2026 Sep 17;22:11637. doi: 10.15766/mep_2374-8265.11637 (PMC13582539; doi:10.15766/mep_2374-8265.11637)
Supplement: Supplementary file 1 [file mep_2374-8265.11637-s001.zip › B. Standard Work.docx]

**Triage Nurse**

**Standard Work Instructions**

Open a “stopwatch app” or utilize your personal watch.

(Synchronize the start of the simulation with the room timekeeper)

**1** – Every 15 seconds (starting at time 0), check a Patient in and assign a “triage acuity” by randomly selecting a Brick from the bag.

a. Low acuity = BLUE Brick
b. High acuity = RED Brick

**2** - Place Patient (Brick) in one of the 2 waiting rooms

If both waiting rooms are full - remove the longest waiting patient (Brick) and place them in the “AMA area”

This represents a patient leaving the ED without being seen (AMA) due to a long wait.

Simulation will run for 5 minutes

| Simulation Reset Needs | 14 Blue Bricks |
| --- | --- |
|  | 6 Red Bricks |
|  | 1 bag for randomization |

**DO NOT TURN PAGE OVER UNTIL INSTRUCTED**

This session is to provide a hands-on, interactive, small group simulation exercise to practice using the scientific problem solving method.

We will have about 2 hours and run the simulation 4 times.

| Simulation Reset Needs | 14 Blue Bricks |
| --- | --- |
|  | 6 Red Bricks |
|  | 1 bag for randomization |

**DO NOT TURN PAGE OVER UNTIL INSTRUCTED**

**ED Nurse**

**Standard Work Instructions**

**3**- Room the Patient (Brick) by adding a GREEN Brick and placing in any open and clean ED Room. (The ED Nurse will obtain a patient (Brick) from Triage.)

- Any Patient with a “high acuity” (RED Brick) MUST be roomed immediately. IF all ED rooms are full, you can move a “low acuity” patient (BLUE Brick) into the hallway. (outside of the ED boxes)

**5**- After labs are ordered, draw labs (only one patient at a time) by adding a GREEN Brick. You must draw labs from any “high acuity” (RED Brick) patient that is roomed in the ED before drawing labs from a “low acuity” patient (BLUE Brick).

**8**- DISCHARGE any patient with discharge orders by placing in the “Discharged” area. Discharged patients will NOT have any RED in the brick tower.

**Place a PINK Brick in the room after discharging the patient to indicate the room is ready to be cleaned**.

| Simulation Reset Needs | 40 Dark Green Bricks |
| --- | --- |

**DO NOT TURN PAGE OVER UNTIL INSTRUCTED**

This session is to provide a hands-on, interactive, small group simulation exercise to practice using the scientific problem solving method.

We will have about 2 hours and run the simulation 4 times.

| Simulation Reset Needs | 40 Dark Green Bricks |
| --- | --- |

**DO NOT TURN PAGE OVER UNTIL INSTRUCTED**

**ED Physician**

**Standard Work Instructions**

**4-** After the ED Nurse has roomed the patient, assess the Patient (Brick) and order labs by adding a YELLOW Brick on top of the brick stack.

**7-** After labs are run decide on disposition of Patient by:

- Admitting Patients with ANY RED Bricks by adding another RED Brick
- Discharging Patients WITHOUT any RED Bricks by adding another BLUE Brick.

| Simulation Reset Needs | 20 Yellow Bricks |
| --- | --- |
|  | 14 Blue Bricks |
|  | 12 Red Bricks |

**DO NOT TURN PAGE OVER UNTIL INSTRUCTED**

This session is to provide a hands-on, interactive, small group simulation exercise to practice using the scientific problem solving method.

We will have about 2 hours and run the simulation 4 times.

| Simulation Reset Needs | 20 Yellow Bricks |
| --- | --- |
|  | 14 Blue Bricks |
|  | 12 Red Bricks |

**DO NOT TURN PAGE OVER UNTIL INSTRUCTED**

**Hospitalist**

**Standard Work Instructions**

**11-** For each patient admitted to the hospital, roll dice until you get

Three 1’s, then Three 2’s, and then One 3.

(The 3 rounds of rolling dice are related to “rounding,” “meeting discharge criteria” and “discharge”).

Once dice roll is complete, place an ORANGE Brick on brick stack to signify discharge orders being placed.

| Simulation Reset Needs | 20 Orange Bricks |
| --- | --- |
|  | 1 dice |

**DO NOT TURN PAGE OVER UNTIL INSTRUCTED**

This session is to provide a hands-on, interactive, small group simulation exercise to practice using the scientific problem solving method.

We will have about 2 hours and run the simulation 4 times.

| Simulation Reset Needs | 20 Orange Bricks |
| --- | --- |
|  | 1 dice |

**DO NOT TURN PAGE OVER UNTIL INSTRUCTED**

**Lab Technician**

**Standard Work Instructions**

**6-** Run the labs once samples from **at least** **two patients** are present by adding a randomly picked BRICK from the bag.

*** Just to reinforce, you must wait until at least 2 patients are present (have 2 GREEN Brick) before running the labs.

a. RED BRICK = Lab result reveals “high acuity”
b. BLUE BRICK = Lab result reveals “low acuity”

| Simulation Reset Needs | 14 Blue Bricks |
| --- | --- |
|  | 6 Red Bricks |
|  | 1 bag for randomization |

**DO NOT TURN PAGE OVER UNTIL INSTRUCTED**

This session is to provide a hands-on, interactive, small group simulation exercise to practice using the scientific problem solving method.

We will have about 2 hours and run the simulation 4 times.

| Simulation Reset Needs | 14 Blue Bricks |
| --- | --- |
|  | 6 Red Bricks |
|  | 1 bag for randomization |

**DO NOT TURN PAGE OVER UNTIL INSTRUCTED**

**Transport**

**Standard Work Instructions**

**10-** Move Patients being admitted from the ED to the Inpatient Unit by adding another RED Brick

place a PINK Brick in the room to indicate that the room is ready to be cleaned.

**12 -** Transport any patient being discharged from the inpatient hospital (ORANGE Brick) out of the hospital and place in “Discharged” area.

place a PINK Brick in the room to indicate that the room is ready to be cleaned.

You can only move ONE patient at a time.

| Simulation Reset Needs | 20 Red Bricks |
| --- | --- |

**DO NOT TURN PAGE OVER UNTIL INSTRUCTED**

This session is to provide a hands-on, interactive, small group simulation exercise to practice using the scientific problem solving method.

We will have about 2 hours and run the simulation 4 times.

| Simulation Reset Needs | 20 Red Bricks |
| --- | --- |

**DO NOT TURN PAGE OVER UNTIL INSTRUCTED**

**Environmental Services**

**Standard Work Instructions**

**9 -** Once a Patient leaves a room (PINK Brick will be placed in the room), clean the room by continuously rolling a die until you get three “1’s”, then remove the Brick to indicate it has been cleaned.

Environmental Services can only clean one room at a time and is

responsible for cleaning both the ED and inpatient rooms.

(The 3 rounds of rolling dice are related to “getting supplies”, “wiping surfaces” and “waiting for surfaces to dry”).

| Simulation Reset Needs | 1 dice |
| --- | --- |
|  | 7 Pink Brick |

**DO NOT TURN PAGE OVER UNTIL INSTRUCTED**

This session is to provide a hands-on, interactive, small group simulation exercise to practice using the scientific problem solving method.

We will have about 2 hours and run the simulation 4 times.

| Simulation Reset Needs | 1 dice |
| --- | --- |
|  | 7 Pink Brick |

**DO NOT TURN PAGE OVER UNTIL INSTRUCTED**

**QI Officer**

**Standard Work Instructions**

- Move any patient moved to the ED Hallway to “Left AMA”. This represents the patient leaving AMA.
- Monitor for rooms being cleaned prior to new patients arriving
  - After every patient discharge, a PINK Brick should be placed in the room. Only **Environmental Services** can **remove** the PINK Brick.
  - If a PINK Brick is NOT placed in a room after a patient leaves OR is taken out of a room by any team member other than Environmental Services, mark this as an Error on the data collection sheet.
- Collect Data:
  - Refer to Data Collection sheet for the defined categories and any additional categories determined by your team
  - For errors:
    - An error is a misplaced Brick (each misplaced Brick = 1 error). Compare to the inspection guide
    - ANY RED Brick in a patient = A **High Acuity** Patient and should be admitted rather than discharged from the ED
    - Monitor for Cleaning Errors and record throughout the simulation.

| Simulation Reset | Data Collection Sheet |
| --- | --- |

**DO NOT TURN PAGE OVER UNTIL INSTRUCTED**

This session is to provide a hands-on, interactive, small group simulation exercise to practice using the scientific problem solving method.

We will have about 2 hours and run the simulation 4 times.

| Simulation Reset Needs | Data Collection Sheet |
| --- | --- |

**DO NOT TURN PAGE OVER UNTIL INSTRUCTED**

**QI Officer**

**Standard Work Instructions**

Collect data on Data Collection sheet

An error is a misplaced Brick (each misplaced Brick = 1 error). For errors, reference photo

Accurately discharged patients

(Author Owned Photo)

**DO NOT TURN PAGE OVER UNTIL INSTRUCTED**

This session is to provide a hands-on, interactive, small group simulation exercise to practice using the scientific problem solving method.

We will have about 2 hours and run the simulation 4 times.

| Simulation Reset Needs | Data Collection Sheet |
| --- | --- |

**DO NOT TURN PAGE OVER UNTIL INSTRUCTED**
